# Biomarkers of ocular allergy and dry eye disease

**DOI:** 10.22336/rjo.2023.42

**Published:** 2023

**Authors:** Mihail Zemba, Mihai-Alexandru Ionescu, Ruxandra Angela Pîrvulescu, Otilia-Maria Dumitrescu, Brănişteanu Daniel-Constantin, Mădălina Radu, Alina-Cristina Stamate, Sînziana Istrate

**Affiliations:** * Department of Ophthalmology, “Carol Davila” University of Medicine and Pharmacy, Bucharest, Romania; ** Department of Ophthalmology, “Dr. Carol Davila” Central Military Emergency University Hospital, Bucharest, Romania; *** ArenaMed Clinic Bucharest, Bucharest, Romania; **** Department of Ophthalmology, “Grigore T. Popa” University of Medicine and Pharmacy, Iaşi, Romania

**Keywords:** dry eye disease, ocular allergy, Sjögren’s syndrome, biomarker, prognostic biomarkers, diagnostic biomarker

## Abstract

The most common disorders of the ocular surface are dry eye disease (DED) and ocular allergy (OA). These conditions are frequently coexisting with or without a clinical overlap and can cause a severe impact on the patient’s quality of life. Therefore, it can sometimes be hard to distinguish between DED and OA because similar changes and manifestations may be present. Atopic patients can also develop DED, which can aggravate their manifestations. Moreover, patients with DED can develop ocular allergies, so these two pathological entities of the ocular surface can be considered as mutual conditions that share the same background. Nowadays, by using different techniques to collect tissue from ocular surfaces, the changes in molecular homeostasis can be detected and this can lead to a precise diagnosis. The article provides an up-to-date review of the various ocular surface biomarkers that have been identified in DED, OA, or both conditions.

**Abbreviations: **DED = dry eye disease, OA = ocular allergy, SS = Sjogren syndrome, TBUT = tear break up time, TFO = tear film osmolarity, AKC = Atopic keratoconjunctivitis, ANXA1 = Annexin 1, ANXA11 = Annexin 11, CALT = Conjunctival associated lymphoid tissue, CCL2/MIP-1 = Chemokine (C-C motif) ligand2/Monocyte chemoattractant protein 1, CCL3/MIP-1α = Chemokine (C-C motif) ligand 3/Macrophage inflammatory protein 1 alpha, CCL4/MIP-1β = Chemokine (C-C motif) ligand 4/Macrophage inflammatory protein 1 beta, CCL5/RANTES = Chemokine (C-C motif) ligand 5 /Regulated on Activation, Normal T cell Expressed and Secreted, CCR2 = Chemokine (C-C motif) receptor 2, CCR5 = Chemokine (C-C motif) receptor 5, CD3+ = Cluster of differentiation 3 positive, CD4+ = Cluster of differentiation 4 positive, CD8+ = Cluster of differentiation 8 positive, CGRP = Calcitonin-gene-related peptide, CX3CL1 C-X3 = C motif -chemokine ligand 1 /Fractalkine, CXCL8 = Chemokine (C-X-C motif) ligand 8, CXCL9 = Chemokine (C-X-C motif) ligand 9, CXCL10 = Chemokine (C-X-C motif) ligand 10, CXCL11 = Chemokine (C-X-C motif) ligand 11, CXCL12 = Chemokine (C-X-C motif) ligand 12, CXCR4 = Chemokine (C-X-C motif) receptor 4, EGF = Epidermal growth factor, HLA-DR = Human leukocyte antigen-D-related, ICAM-1 = Intercellular adhesion molecule 1, IFN-γ = Interferon-gamma, IgG = Immunoglobulin G, IgE = Immunoglobulin E, IL-1 = Interleukin-1, IL-1α = Interleukin-1 alpha, IL-1β = Interleukin-1 beta, CGRP = Calcitonin-Gene-Related Peptide, IL-3 = Interleukin-3, IL-4 = Interleukin-4, IL-6 = Interleukin-6, IL-8 = Interleukin-8, IL-10 = Interleukin-10, IL-17 = Interleukin-17, IL-17A = Interleukin-17A, LPRR3 = Lacrimal proline-rich protein 3, LPRR4 = Lacrimal proline-rich protein 4, MUC5AC = Mucin 5 subtype AC, oligomeric mucus/gel-forming, MUC16 = Mucin 16, OCT = Optical coherence tomography, OGVHD = Ocular graft versus host disease, PAX6 = Paired-box protein 6, VKC = Vernal keratoconjunctivitis, TGF-β = Transforming growth factor β, S100 = proteins Calcium activated signaling proteins, Th1 = T helper 1 cell, Th17 = T helper 17 cell, MGD = Meibomian gland dysfunction, TFOS = Tear film and ocular surface society, SS-KCS = Keratoconjunctivitis Sicca, MMP-9 = Matrix metalloproteinase 9, MMP-1 = Matrix metalloproteinase 1, ZAG = Zinc alpha glycoprotein, CBA = Cytometric bead array, MALDI TOF-MS = matrix assisted laser desorption ionization-time of flight, SELDI TOF-MS = surface-enhanced laser desorption ionization-time of flight, IVCM = in vivo confocal microscopy, AS-OCT = anterior segment optical coherence tomography, iTRAQ = Isobaric tags for relative and absolute quantitation, LC-MS = Liquid chromatography-mass spectrometry, LCN-1 = lipocalin 1, PIP = prolactin induced protein, NGF = Nerve growth factor, PRR4 = proline rich protein 4, VIP = Vasoactive intestinal peptide, ELISA = enzyme linked immunoassay, TNF-α = tumor necrosis factor alpha, PAC = perennial allergic conjunctivitis, SAC = seasonal allergic conjunctivitis, IC = impression cytology, RT-PCR = reverse transcription polymerase chain reaction, PCR = polymerase chain reaction, APCs = antigen-presenting cells, NK cells = natural killer cells, HEL = hexanoyl-lysine, 4-HNE = 4-hydroxy-2-nonenal, MDA = malondialdehyde

## Introduction

**Fig. 1:**
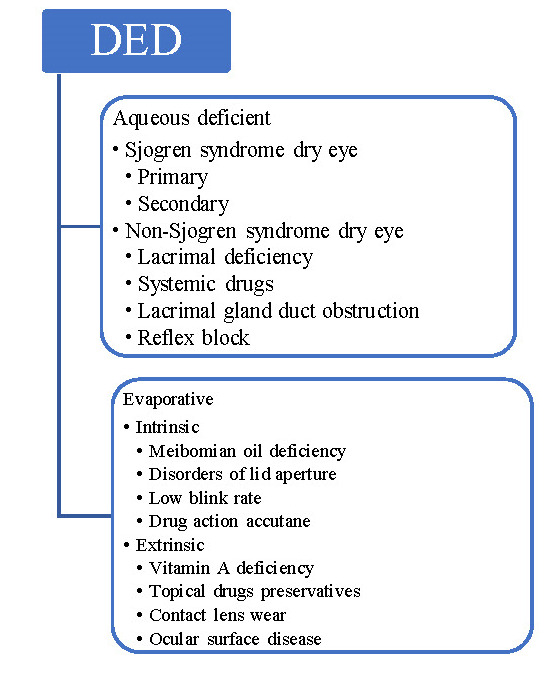
Classification of DED [**3**]

DED is a disorder of the ocular surface characterized by changes in the tear film homeostasis, inflammation with or without significant injuries, and atypical neurosensorial manifestations [**1**]. DED can be classified into two categories based on the mechanism of action: aqueous-deficient characterized by a reduction in the lacrimal gland function and evaporative characterized by Meibomian gland dysfunction [**2**]. Withal, aqueous-deficient DED can be classified as SS-dry eye and NonSS-dry eye, and evaporative DED can be classified as Intrinsic and Extrinsic [**2**]. The classification of DED is illustrated in **Fig. 1**. The evaporative form of DED is more common, but most of the patients can present both forms [**2**,**3**]. 

There are many factors implicated in DED, most of which are environmental factors like UV radiation [**4**] and ozone [**5**]; also, the chronic use of glaucoma eyedrops can modify the ocular surface homeostasis due to the preservative content [**6**]. 

The prevalence of DED is the highest in Southeast Asia and lowest in Europe. Moreover, women are more predisposed than men and the elderly develop DED more frequently than young people [**7**].

At present, DED can be diagnosed using the patient’s symptomatology, medical history, and some objective tests that measure the ocular surface integrity and/or tear function. The most commonly used tests are Schirmer’s test, fluorescein TBUT, tear film osmolarity, and staining of ocular surface epithelia (**Table 1**) [**8**,**9**].

**Table 1:** Diagnosis of dry eye disease [**8**,**9**]

Screening tests	Symptomatology
	Risk factors
Diagnostic tests	**Routine tests**: Schirmer's test, TBUT, TFO, ocular surface staining
	**Additional tests**: tear film analysis, electrophoresis of tear film proteins, interferometry, meibometry, thermography, flow citometry, confocal microscopy
Subtype Classification tests	Tear volume tests
	Lipid thickness
	Meibomian gland dysfunction imaging

On the other hand, OA is a very common condition with a 20% prevalence worldwide, which contains a collection of hypersensitivity disorders that affect the eyelid, conjunctiva, and cornea [**8**,**9**]. The most common type of OA is allergic conjunctivitis. It is a condition characterized by an IgE-mediated response of the ocular surface to different allergens, such as pet dander, diverse types of pollens, or dust mite dander [**10**]. 

Even if DED and OA are different ocular surface pathologies, there was a lot of evidence of shared pathogenic pathways between these conditions. There are at least three different possible scenarios: a) DED and OA are present concomitant, b) a patient with DED develops OA, and c) a patient with OA develops DED [**10**]. 

Biomarkers are indicators of normal or pathogenic processes and can also measure the response to an exposure or after a therapeutic intervention [**11**]. Including eye diseases, an efficacious biomarker that can indicate ocular surface abnormalities can be measured and collected from the cornea, tears, and conjunctiva.

## Materials and methods

We conducted a comprehensive literature search in the MEDLINE electronic database using the PubMed interface. The word combinations used in the search process were “dry eye disease biomarkers”, “ocular allergy biomarkers”, and “ocular surface biomarkers”. Inclusion criteria consisted of articles written in English and regarding human pathology that were published before December 2022. Additional references were selected from the reference lists of the already retained studies. We excluded duplicates, studies not relevant to the topic, studies on animal models, as well as conference presentations, editorials, letters to editors, and comments. After applying the inclusion and exclusion criteria, we retained 134 articles dating from 1994 to 2022.

## Results

*Corneal biomarkers*

The cornea is a transparent avascular tissue, composed of five layers, and one of the most innervated tissues in the body [**12**]. Along with the tear film, the cornea makes up the anterior refractive surface of the eye, representing two-thirds of the total refractive power [**13**]. 

*In vivo confocal microscopy*

IVCM is the method used to study the corneal subbasal nerve plexus [**14**]. Some elements evaluate nerve alterations: total, trunk, and branch nerve density and length, tortuosity, beading, nerve reflectivity and thickness, and microneuromas [**15**]. IVCM is more commonly employed in the study of nerve density and length in DED [**16**], herpes simplex keratitis [**17**], and postrefractive surgery [**18**]. However, other patients with anterior segment disorders, such as keratoconus [**19**], vernal [**20**], and atopic keratoconjunctivitis [**21**] have also been investigated using IVCM. 

In most ocular and systemic conditions, studies reported decreased corneal nerve density and length [**15**]. Studies have shown conflicting results regarding nerve density and corneal sensation in DED [**22**]. Therefore, nerve density was negatively correlated with symptom severity [**23**] and positively correlated with corneal sensations in DED [**22**]. Furthermore, nerve density can be used as a predictive biomarker for the treatment response because patients with a higher nerve density and DED responded better to the treatment [**24**]. Also, IVCM showed nerve regeneration in DED patients after treatment, suggesting that this parameter can be used as a pharmacodynamic biomarker [**24**]. 

Regarding nerve tortuosity in DED, many studies reported a positive correlation with corneal sensation and a negative correlation with fluorescein stain and Schirmer’s test [**23**,**25**-**27**]. Increased nerve tortuosity was reported [**23**,**25**] without a significant correlation with signs and symptoms [**23**]. Despite that, it could be concluded that tortuosity might be used as a pharmacodynamics biomarker because most of the studies showed decreased nerve tortuosity after the treatment [**24**]. 

Studies showed no significant results about nerve reflectivity and thickness in DED [**15**] and referring to nerve beading, an increase was reported in several studies [**23**,**25**]. Microneuromas, which have been considered the source of spontaneous excitations in postsurgical pain, need more validation studies to be considered as biomarkers [**18**,**28**,**29**].

To summarize, the most validated parameters are nerve fiber density and length. Other nerve parameters necessitate more validation and standardization due to their variability. Probably, the nerve density and length used as a single diagnostic biomarker is not so useful, but it can monitor the level of nerve damage and can be correlated with other nerve parameters helping to establish the correct diagnosis [**30**].

*Corneal thickness*

The AS-OCT is a non-invasive and non-contact method used to evaluate epithelial corneal thickness. Recently, epithelial thickness has been used as a diagnostic biomarker for different conditions [**31**,**32**]. For example, in DED and limbal stem deficiency patients, studies have shown thinning of the epithelial thickness compared to controls [**33**]. Epithelial thickness measured by the AS-OCT has the potential to be a biomarker in some ocular surface conditions even if it has a promising role in the diagnosis of keratoconus [**34**]. 

*Tear film*

*The tear film - definition, composition*

A stable tear film is defined as a collection of fluids made of two important components: a muco-aqueous layer and a lipid layer [**35**]. The muco-aqueous layer contains a diversity of glycoproteins combined with an aqueous layer that maintains a proper osmolarity and the lipid layer contains a lot of lipids that prevent the loss of the aqueous layer [**35**]. Tears are formed by thousands of different molecules secreted by lacrimal and Meibomian glands, goblet, nerve, and ocular surface cells [**36**]. 

There are four types of tears based on the mechanism of production: 

1) basal tears, which cover the eye constantly and are indispensable for a healthy ocular surface. This type of tear is deficient in DED and autoimmune diseases. 

2) reflex tears - produced due to the sneeze reflex or nasal stimulation.

3) closed eye tears - produced during the sleep period, useful in maintaining the ocular surface homeostasis. 

4) emotional tears - formed due to different emotions [**35**]. 

*Collection of tears*

Using different techniques, the content of tears can be studied and some biomarkers can be isolated and used for the diagnosis of different pathologies among which are DED and OA. A lot of non-invasive methods are used to collect tears and, some conditions must be respected. For example, it is very important not to activate the corneal nerves during collection because reflex tearing is induced, which can alter the tear’s composition, and the use of artificial tears and topical anesthesia can have an impact on the tear composition [**36**]. 

Different technologies such as DropArray and CBA-Luminex can analyze numerous molecules only by using a small volume of tears [**37**]. In addition, using different mass spectrometric techniques, including SELDI-TOF-MS and MALDI-TOF-MS, the content of tears proteomics can be closely analyzed. iTRAQ technology has also enhanced the accuracy in the evaluation of the tear’s protein mass [**37**-**39**]. 

Biomarkers from tears used to diagnose DED or OA can be classified as: 

- protein biomarkers; 

- inflammatory cytokines/chemokines; 

- tear osmolarity.

*Tear biomarkers - Proteins*

The tear proteome is formed by approximately 2000 proteins and lipocalin, albumin, lactoferrin, and IgA representing about 70-80% of the protein content [**40**]. One of the most important protein biomarkers is lactoferrin, which plays an important role in ocular inflammatory response and cell growth. Low levels of lactoferrin have also been reported in different conditions such as aqueous deficient DED, and herpes simplex keratitis [**41**]. Moreover, the role of lactoferrin and lysozyme are known to be antibacterial, while lipocalin is the major lipid-binding protein in tears. In a literature study that investigated the change of protein content of tears in patients with non-SS, SS DED, and SJ-syndrome, different proteins, such as lysozyme C, lipocalin 1, lactoferrin, and LPRR3, LPRR4 were found decreased compared to normal population [**42**-**44**]. In DED, the use of i-TRAQ quantitative proteomics demonstrated reduced tear levels of four proteins, LCN-1, PIP, lactoferrin, and lysozyme [**45**]. Another important protein biomarker found in tears is the S100 protein family. These are calcium-binding proteins, which play a proinflammatory role and seem to be downregulated in DED patients [**46**,**47**]. Also, using ELISA methods, intracellular proteins such as ANXA1, clusterin, and ANXA11 were found in the tear content and used as potential biomarkers. Moreover, they are downregulated in patients with DED [**48**,**49**]. However, some studies identified protein biomarkers that have been upregulated in DED patients. For example, the levels of α-enolase in the tear content can be an important diagnostic tool for dry eye patients in 85% of cases [**45**]. 

Members of the mucin family and Cathepsin S protein have been also investigated as protein biomarkers in the tear content [**50**,**51**]. MUC5AC is a significant mucin family protein with a role in lubrication and hydration of mucosal surfaces. Some studies have shown a decreased amount of MUC5AC in both SS-DED and nonSS-DED patients and these are also correlated with increased inflammation [**52**-**54**]. Cathepsin S is a lysosomal cysteine peptidase involved in immune responses, which has significant activity in tears of SS-DED patients [**53**-**54**]. Neuromediators, such as VIP, CGRP, and NGF have been investigated in tears too and it has been found that in DED patients the NGF amount was increased, while the CGRP amount was decreased [**55**-**57**].

Regarding OA, there is a limited number of available studies that pointed out several potential biomarkers in tears. So, proteins like lactoferrin, IgA, and eosinophil neurotoxin were found to be increased in OA tear patients [**58**-**61**]. Also, serum albumin and α-defensin were found to be upregulated in OA patients [**62**,**63**]. In a literature study, the authors reported conflicting results about tear concentration of ZAG and lipocalin-1 in OA patients [**64**,**65**]. The explanation may be due to demographic factors or differences between the analysis techniques, but more research is warranted to determine the concrete biomarkers and their role in OA [**66**]. 

*Tear biomarkers – Inflammatory cytokines/chemokines*

Cytokines and chemokines are endogenous inflammatory molecules produced by a variety of cells that can also be found in human tears [**67**]. Different studies reported that in inflammatory conditions such as DED, OGVHD, SS, and OA, these molecules are increased [**37**]. IVCM can be used to monitor and quantify the level of ocular surface inflammation. An important mechanism in inflammatory ocular surface response is played by the imbalance of metalloproteinases and their inhibitors [**21**,**68**-**71**]. MMP-9 and MMP-1 are proteinases expressed by eosinophils, involved in corneal and conjunctival remodeling processes and their activity at the ocular surface level is significantly higher in OA patients. Also, some studies described a large amount of MMP-9 in the tears of DED and OGVHD patients [**72**]. 

IFN-γ is a cytokine secreted by a series of inflammatory cells such as T-helper 1 cells, NK cells, and epithelial cells and is thought to be a representative biomarker of the inflammatory status of the ocular surface [**73**]. High levels of IFN-γ were reported in tears in patients with DED and OA [**74**]. Increased levels of proinflammatory cytokine IL-1 have been detected in the tears of DED patients [**67**]. High levels of TNF-α and IL-1 were also found in the tears of patients with OA [**75**]. Other important inflammatory biomarkers found in tears in patients with DED and OA are IL-17, IL-22, and IL-6, which are the effector cytokines of Th-17 cells. The levels of these cytokines are increased in SS-DED patients and non-SS-DED patients compared to normal [**76**-**79**]. Moreover, IL-6 has pro and anti-inflammatory roles and is a representative biomarker of treatment effects [**80**]. IL-8 is another key chemokine that has been consistently increased in DED patients [**81**]. A literature study reports that IL-8, IL-2, IFN-γ, and EGF may represent biomarkers of disease gravity in DED [**73**]. Several studies reported that the levels of CXCL chemokine family and macrophage inflammatory proteins chemokine family were higher in SS-DED patients compared to nonSS-DED patients [**82**,**83**].

Regarding OA, some inflammatory biomarkers stand out. Using a non-invasive method, IgE can be highlighted in VKC, SAC, and PAC patients compared to normal subjects [**84**,**85**]. In another literature study, the levels of IL-5 and TNF-α in AKC patients were notably higher compared to the eyes of controls [**86**].

*Tear biomarkers – Tear osmolarity*

Different factors, such as increased tear evaporation, and modifying protein and electrolyte content in tears, cause the modification of tear film osmolarity. A difference of > 8mOsm/L between eyes is proposed to be suggestive of DED and also, a range of osmolarity of 308mOsm/L to > 316mOsm/L is used as a cut-off diagnostic for DED [**36**].

*Conjunctiva*

*Structure and function*

The conjunctiva is a thin mucous membrane that protects the sclera, having the function of maintaining the ocular surface homeostasis. It is formed of palpebral, forniceal, and bulbar conjunctiva and consists of epithelium cells, goblet cells, and connective tissue [**87**]. Moreover, it contains lymphoid tissue, lacrimal accessory glands, and mast cells. The goblet cells secrete MUC5AC [**88**,**89**], a mucin that is in the middle of many ocular conditions, and the CALT (lymphoid tissue of conjunctiva) is responsible for the immune response [**90**]. During ocular surface inflammation present in DED or OA, proinflammatory modulators such as TNF-α, IL-6 intercellular adhesion molecules such as ICAM-1, and various types of immune cells are found in the epithelial conjunctival layer [**88**]. 

*Collection of Conjunctival cells*

One of the most common methods used to analyze the biomarkers of conjunctiva is impression cytology. It is a well-known minimally invasive and easily repeatable technique [**91**,**92**]. A complementary approach to IC to gather different region conjunctival cells is brush cytology [**93**]. A more invasive technique is the collection of conjunctival samples by excision of conjunctival tissue. Using various analytical methodologies such as microscopy, immunohistochemistry, flow cytometry, and RT-PCR, biomarkers from the collected sample can be determined [**93**,**94**]. Moreover, another noninvasive diagnostic tool used in different ocular disorders to collect conjunctival biomarkers is in vivo confocal microscopy [**95**]. 

*Biomarkers in the conjunctiva - Inflammatory biomarkers*

IVCM is one of the methods used to identify proinflammatory cytokines in the conjunctiva [**71**,**96**-**98**]. Different types of inflammation molecules, such as TNF-α, IFN-γ, and IL-1α, are overexpressed in the ocular surface of DED and OA patients [**98**-**100**]. Moreover, cytokines considered specific to allergy, IL-4, and IL-5 are overexpressed in the ocular surface of DED patients too and another key inflammatory biomarker that can be evidenced in the conjunctiva of DED patients is IL-17 secreted by Th-17 cell [**101**,**102**]. In a literature study that compares the conjunctival cytology of ten patients with DED and ten control patients, the level of proinflammatory cytokines mentioned above was significantly higher in DED patients compared to controls [**100**-**103**]. Also, the CCR5 receptor was evidenced in the conjunctiva of patients with both forms of DED [**103**,**104**]. Another method used to identify inflammatory biomarkers in the conjunctiva is immunohistochemistry. Thus, DED patients can present an infiltrate of T cells (CD3+, CD4+) in the connective tissue structure of the conjunctiva [**102**-**105**].

Therefore, oral antihistamines can represent an additional contribution to DED in atopic patients [**86**,**106**-**109**]. 

All these chemokines can determine epithelial cell differentiation, squamous cell metaplasia, and goblet cell apoptosis and these may facilitate allergic reactions and DED development [**86**,**106**-**109**].

*Biomarkers in the conjunctiva - Protein markers*

An important immune glycoprotein found in conjunctival epithelial cells is HLA-DR [**110**]. The levels of conjunctival HLA-DR in DED are higher compared to normal subjects and also, a relationship between DED severity and HLA-DR expression can be found [**111**,**112**]. PAX6 is a well-known protein that evaluates ocular surface damage. Downregulation of PAX6 in DED-SS patients was highly associated with ocular surface damage [**109**-**113**]. ICAM-1 is found in various cells and it is upregulated in response to diverse inflammatory processes and oxidative stress [**114**,**115**]. Moreover, the role of galectin and β-galactoside has been demonstrated in OA allergy. The levels of these proteins are increased in VKC patients compared to controls [**113**-**116**].

*Biomarkers in the conjunctiva - Lipid markers*

Oxidative stress plays an important role in different ocular surface disorders. The oxidative damage can be detected by measuring the end products of lipid peroxidation [**114**-**117**]. The biomarker that can be highlighted is HEL, which is an early-phase marker of lipid peroxidation, and 4-HNE, MDA, which are late-phase markers [**118**]. Also, the levels of different radicals, such as 4-hydroxy-2-noneal and malondialdehyde in ocular surface were raised in DED patients compared to controls [**96**,**117**-**119**]. Moreover, endogenous steroids can affect the lipids secreted by Meibomian glands and they were investigated as markers for DED. So, a decreased level of cortisol and androstenedione was detected in DED patients [**96**,**119**].

*Biomarkers in the conjunctiva – Goblet cells and mucins*

Mucins are glycoproteins that act as a mucosal barrier, crucial in ocular surface protection. The most important mucins of glycocalyx are the transmembrane mucins [**117**-**121**]. Mucins present in conjunctival tissue belong to three categories: a) mucins formed by goblet cells, b) soluble mucins, and c) membrane-associated mucins that are present in the epithelial cells [**118**-**122**]. MUC5AC is the main secreted mucin and is present in tears too. It is well known that is down-expressed both in the ocular surface of DED and OA patients [**123**].

Goblet cells are responsible for lubrication, ocular surface wetting, and prevention of infection [**117**-**119**]. Several literature studies revealed that a decrease in goblet cell density affects mucin secretion and occurs in ocular surface inflammation disorders [**119**,**120**]. Due to the presence of inflammation, the cornea and conjunctiva are damaged and a depletion of goblet cells occurs in DED, the result being a deficiency of mucins [**115**-**120**]. An increase in goblet cell density may be an indicator of a healthy eye surface and can represent a biomarker of treatment effect [**120**-**123**].

Goblet cells are also under parasympathetic nervous system control and ocular surface inflammation alters their function causing a vicious circle characterized by their depletion and loss of mucins [**122**,**123**].

## Conclusions

Corneal biomarkers can be useful in pharmacodynamics studies, but more studies are needed to accurately determine their role in the diagnosis of DED or OA. Regarding the study of tear content, proteins are among the most important biomarkers, especially lactoferrin. Correlating signs and symptoms, TBUT, and Schirmer’s test result with the lactoferrin tear’s value, a correct differential diagnosis between DED and OA can be done. 

Inflammation plays a crucial role in the development of DED and OA. Even if some specific inflammatory biomarkers are used to differentiate DED from OA, these can be present in both conditions, making the differential diagnosis more difficult. For example, the proinflammatory cytokines specific to OA, IL-4, IL-5, and IL-13, are also overexpressed in tears of DED patients. Furthermore, the glycoproteins known as mucins that are present on the ocular surface can be useful biomarkers in the diagnosis of DED or OA. MUC5AC, the main ocular surface mucin, is downexpressed in both DED and atopic patients. 

It can be concluded that the study of biomarkers in ocular matrices improved our knowledge about the pathophysiology of diseases, helped in the correct diagnostic of different conditions, and, biomarkers have been useful in exploring treatment development and efficacity. 

**Conflict of Interest statement**

The authors declare no conflict of interest.

**Informed Consent and Human and Animal Rights statement**

Not applicable.

**Authorization for the use of human subjects**

Not applicable.

**Acknowledgments**

None.

**Sources of Funding**

This research received no external funding.

**Disclosures**

None.
